# SYT-SSX1 enhances the invasiveness and maintains stem-like cell properties in synovial sarcoma via induction of TGF-β1/Smad signaling

**DOI:** 10.1186/s12885-022-09229-5

**Published:** 2022-02-12

**Authors:** Yan Qi, Shuang-Shuang Dong, Yong-Lai He, Zi-Han Liu, Ya-Lan Huang, Ning Wang, Zhen Zhang, Zhong Li, Mei Er Tu He Ta Mi Shi, Xiao Feng, Qing Yao, Hong Zou, Jian-Ming Hu, Li-Juan Pang, Feng Li

**Affiliations:** 1 Department of Pathology, Central People’s Hospital of Zhanjiang & Zhanjiang Central Hospital, Guangdong Medical University, Zhanjiang, Guangdong 524000 China; 2 Department of Pathology, Shihezi University School of Medicine & the First Affiliated Hospital to Shihezi University School of Medicine, North 2 road, Shihezi, Xinjiang, 832002 China; 3 Department of Pathology, Northern Jiangsu People’s Hospital Affiliated to Yangzhou University/Clinical Medical College, Yangzhou University, Yangzhou, Jiangsu 225000 China; 4 Department of ICU, Central People’s Hospital of Zhanjiang & Zhanjiang Central Hospital, Guangdong Medical University, Zhanjiang, Guangdong 524000 China; 5 Department of Pathology, The Affiliated Hospital of Qingdao university, Qingdao, China; 6 Department of Pathology, Suining Central Hospital, Suining, Sichuan China; 7 Department of Pathology, Beijing Chaoyang Hospital, Capital Medical University, Beijing, China

**Keywords:** SYT-SSX1, Cancer stem cell, Synovial sarcoma, TGF-β1/Smad, Signaling pathway, Epithelial–mesenchymal transition

## Abstract

**Background:**

Synovial sarcoma (SS) is a type of soft tissue sarcoma (STS) of undetermined tissue origin, which is characterized by the recurrent pathognomonic chromosomal translocation t (X;18)(p11.2; q11.2). Studies have shown that SS is a malignant tumor originating from cancer stem cells or pluripotent mesenchymal stem cells and may be related to fusion genes. In addition, some studies have indicated that the induction of epithelial–mesenchymal transition (EMT) via the TGF-β1/Smad signaling pathway leads to SS metastasis.

**Methods:**

We analyzed the effects of SYT-SSX1 on the stemness of SS cells via TGF-β1/Smad signaling in vitro. The *SYT-SSX1* fusion gene high expression cell was constructed by lentiviral stable transfer technology. SYT-SSX1 and SW982 cells were cultured and tested for sphere-forming ability. The transwell migration assay and flow cytometry were used to assess the migration ability of the sphere cells as well as the expression of CSC-related markers. We treated SYT-SSX1 cells with rhTGF-β1 (a recombinant agent of the TGF-β1 signaling pathway) and SB431542 and observed morphological changes. A CCK-8 experiment and a western blot (WB) experiment were conducted to detect the expression of TGF-β1 signaling pathway- and EMT-related proteins after treatment. The SYT-SSX1 cells were then cultured and their ability to form spheres was tested. Flow cytometry, WB, and quantitative real-time polymerase chain reaction (qRT-PCR) were used to detect the expression of CSC surface markers on SYT-SSX1 sphere cells.

**Results:**

It was found that SYT-SSX1 has stronger sphere-forming ability, migration ability, and higher expression of CSC-related molecules than SW982 cells. Through treating SYT-SSX1 and SW982 cells with rhTGF-β1 and SB431542, we found that TGF-β1 enhanced the proliferation of cells, induced EMT, and that TGF-β1 enhanced the characteristics of tumor stem cells.

**Conclusions:**

Our results suggest that *SYT-SSX1* enhances invasiveness and maintains stemness in SS cells via TGF-β1/Smad signaling. These findings reveal an effective way to potentially improve the prognosis of patients with SS by eliminating the characteristics of cancer stem cells (CSCs) during treatment.

**Supplementary Information:**

The online version contains supplementary material available at 10.1186/s12885-022-09229-5.

## Background

Synovial sarcoma (SS) is a highly aggressive malignant tumor of mesenchymal origin and is the fourth most common type of soft tissue sarcoma (STS), accounting for approximately 5–10% of all STSs [1]. SS was still considered to be of uncertain histological origin in the 2016 WHO soft tissue classification, particularly as it exhibits a biphasic histopathological form in terms of morphology and immunohistochemical phenotype. However, recent studies have shown that SS may originate from cancer stem cells (CSCs) or pluripotent mesenchymal stem cells [2, 3]. Patients with SS have a poor prognosis (5-year survival rate: 50% [4], 10-year survival rate: 10–30% [5]), as it is prone to local recurrence and distant metastasis. The current clinical treatment of SS has not significantly progressed over the past 20 years owing to a lack of targeted therapies. Surgical resection is the first choice for treatment followed by postoperative adjuvant radiotherapy and chemotherapy [5, 6].

SS is characterized by the specifically aberrant chromosomal translocation t(X;18)(p11.2; q11.2) that generates the *SYT-SSX* fusion gene (including *SYT-SSX1*, *SYT-SSX2*, or rarely *SYT*-*SSX4*). The resulting oncogenic fusion proteins play critical roles in the oncogenesis and development of SS [7]. Currently, studies have indicated that SYT-SSX may be related to histological subtype [8]. Saito et al. suggested that almost all biphasic SS (BSS—has sufficient capacity for epithelial differentiation to form glandular structures) harbor the *SYT-SSX1* fusion gene [9]. This indicates that fusion genes may be associated with epithelial–mesenchymal transition (EMT). Naka et al. suggested that SS was a stem cell malignancy caused by the SS18-SSX fusion protein as it had the capacity for self-renewal and multi-lineage differentiation, which had the ability to induce EMT [2, 3].

Accumulating evidence indicates that CSCs may be a critical driving force for several types of cancers. Doherty et al. proposed that non-malignant cells become malignant stem cells after EMT [10]. Furthermore, EMT-derived tumor cells acquire stem cell properties and exhibit marked therapeutic resistance [11–13]. The expression and activation of EMT-related transcription factors occurs in several signaling pathways, including the transforming growth factor β (TGF-β), bone morphogenetic protein (BMP), Wnt, Notch, and Hedgehog pathways [14]. The TGF-β/Smad pathway is the classic signaling cascade that induces EMT, and results in characteristic outcomes similarity to those observed in cancer stem cells in gastric carcinoma, ovarian clear cell carcinoma, and non-small cell lung cancer [15–21]. However, there are few studies on the relationship between sarcoma and CSC, particularly in SS.

Herein, our study confirmed that SS is a stem cell-derived malignant tumor, and SYT-SSX1 can induce SS cells to participate in EMT transformation as well as enhance the activity of SS stem cells through the TGF-β1/Smad signaling pathway.

## Materials and methods

### Cell lines

SW982 human synovial sarcoma cells were purchased from the Shanghai Institute of Biochemistry and Cell Biology (ATCC, HTB-93™). The SW982 cell line was derived from a biphasic SS removed from a 25-year old woman as previously described [22]. Cells were grown in L-15 medium containing 10% fetal bovine serum (FBS; Gibco, CA, USA), and 1% penicillin/streptomycin (Gibco), in a 37 °C incubator with humidified atmosphere of 5% CO_2_.

### Antibodies

Primary antibodies used in this study included rabbit anti-SOX-2 (1:1000 dilution, Abcam, Cambridge, UK), rabbit anti-Nanog (1:1000 dilution, CST, Danvers, Massachusetts, USA), rabbit anti-OCT-4 (1:1000 dilution, Abcam), rabbit anti-pSmad2/3 (1:500 dilution, Abcam), rabbit anti-Snail (1:500 dilution, Abcam), rabbit anti-E-cadherin (1:500 dilution, Abcam), rabbit anti-TGF-β1(1:1000 dilution, Abcam), rabbit anti-N-cadherin (1:1000 dilution, Wanleibio), rabbit anti-Slug (1:1000 dilution, Wanleibio) and mouse anti-β-actin (1:1000 dilution, ZSGB-BIO, Beijing, China).

### Lentivirus construction

Lentivirus carrying the *SYT-SSX1* fusion gene (with green fluorescent label) was purchased from Shanghai Genechem. First, linear vector was obtained by restriction enzyme digestion. Carrier information: carrier name: GV358, sequence of components: ubi-mcs-3flag-sv40-egfp-res-puromycin, cloning site: AgeI/AgeI, fluorescent labeling: EGFP. Next, primers specific to the target gene, *SYT-SSX1*, were used to amplify it using polymerase chain reaction (PCR). Then the target gene amplification product was ligated into the linear GV358 to create a new vector. After the recombined vector was transformed into bacteria, PCR was used to positively identify the amplified target gene sequence. Positive PCR products were selected for sequencing, and the bacteria containing the new vector were cultured based on the results, so as to obtain high-purity target gene plasmids suitable for lentivirus packaging. With high purity plasmid transfection 293 T cells, and join the viral vector DNA mixture, at 37 °C and 5% CO2 incubator. According to the cell state, supernatant was collected, supernatant was centrifuged and discarded. After removing cell debris, virus preservation solution was added and resuspended to detect lentivirus quality. High-quality lentivirus was selected for the cell transfection experiment.

### Lentivirus transfection pretest (96-well plate)

Three treatment groups were established with MOIs of 100, 10, and 1, respectively. The green fluorescence expression was observed in the three groups after the virus concentration was mixed with different media. The medium was divided into four groups, namely, L-15 medium, L-15 medium+Polybrene, L-15 medium+ENi. S, L-15 medium+Polybrene+ENi.

Based on the green fluorescence expression of lentivirus transfection, the most suitable medium was selected, and the virus concentration MOI in treatment groups were set at 100, 80, 60, and 40, respectively. The most suitable medium was used for transfection, and the most appropriate lentivirus transfection concentration was selected according to the expression of green fluorescence and the cell state for the formal lentivirus transfection experiment.

### Lentiviral transduction experiment (6-well plate)

One day before transduction of SYT-SSX1 lentivirus, 2 × 10^5^ cells/well were seeded in a 6-well plate and incubated until the cell confluence reached 30–40%. The following day, the medium was replaced with fresh L-15 medium, and 5 μg/mL Polybrene (100 μL), L-15 medium (840 μL), and 1 × 10^8^ TU/ml virus fluid (60 μL) were combined. The transfected cells were incubated at 37 °C for 8–12 h, the medium was replaced with fresh L-15 medium, and cells were incubated for Obvious green fluorescence expression was observed 48 to 72 h after transduction. For 72–96 h transduction, stable cell lines were constructed by screening with 0.5 μg/mL puromycin.

### Transwell migration and invasion assay

The day before the experiment, the Matrigel matrix was liquefied overnight in a refrigerator at 4 °C. Additionally, 200 μL pipette tips were placed in the refrigerator at − 20 °C for half an hour before the experiment, and the original medium was pre-cooled to 4 °C. Matrigel and L-15 medium were combined at a ratio of 1:8 to form a diluted Matrigel. Then, 40 μL of the diluted Matrigel was added to the upper chamber of the migration chamber (pore size, 8 μm; Corning, USA) and incubated at 37 °C for 2 h. After the diluted Matrigel had solidified, the remaining liquid in the chamber was drained, and 100 μL of the L-15 original medium was added to each upper chamber, followed by incubation (at 37 °C) for 30 min. To seed the cells, the culture medium was removed from the upper chamber of the small insert and 200 μL of the diluted cell suspension (6 × 10^3^ cells) was added to each well. Then, 600 μL of L-15 medium containing 20% FBS was added to the lower chamber of the 24-well plate. Cells were incubated at 37 °C in a normal humidified atmosphere and allowed to migrate and invade through the Matrigel for 24 and 48 h, respectively. To fix the cells, the chamber was rinsed gently with phosphate-buffered saline (PBS) twice, 600 μL of 4% paraformaldehyde was added to the lower chamber and allowed to fix at 4 °C for 20 min. Inserts were then blow-dried upside down. Next, 600 μL of 0.1% crystal violet was added to the lower chamber for 15 min, and then the inserts were washed twice with PBS. The upper layer of the non-migrated cells was gently wiped off with a cotton swab. The number of cells that migrated or invaded were counted in 5 random fields under a microscope at 100× magnification (OLYMPUS IX71, OLYMPUS, Japan).

### Wound healing assay

The 6-well plate, marker pen, ruler, and micropipette tips were sterilized for 30 min using a UV lamp. The bottom of the 6-well plate was placed upward, and a straight line was drawn on the bottom surface with a marker at intervals of 0.5–1 cm. Approximately 5 × 10^5^ cells/well were seeded onto a 6-well plate and grown until cells reached 90% confluency. Then, the cell monolayer was wounded by introducing a scratch using a sterile micropipette tip along the drawn line. Cells were gently washed with PBS three times to discard the floating cells. Serum-free L-15 medium was added to each well. The cells were incubated at 37 °C in a cultivation box and photographed at 0, 24, and 48 h using microscopy. The measured the area of the scratched area, and 0 h was defined as the benchmark.

### Tumor sphere formation culture

Cell lines were plated at a density of 1 × 10^5^ cells/well in low adhesion 6-well plates (Corning), in serum-free medium L-15 (Gibco) supplemented with B-27 Supplement (1:50; Gibco), growth factors human EGF (20 ng/mL; 10 μL; PeproTech, Rocky Hill, NJ, USA), human bFGF (20 ng/mL; 10 μL; PeproTech), and 1% penicillin/streptomycin (Gibco) to avoid cell aggregation. In addition, the medium was changed every 3 days. After 6 days, the cultured medium from each well was placed into a 15-mL centrifuge tube, flicked, and then 50 μL of cells was pipetted into 5 wells of a 96-well plate; this was repeated three times. The number of sphere cells in each well was then counted using microscopy. The average number of sphere cells was calculated, converted to the same volume, the total number of sphere cells was calculated, and then the rate of sphere formation was analyzed. The remaining cells in the centrifuge tube were centrifuged, the supernatant was discarded, and the pellet was incubated with 0.25% trypsin-EDTA (Life Technologies) for 5 min, the sphere cells were digested into single cell, and the digestion was terminated after 5 min, and cells were centrifuged at 800 rpm for 5 min. The number of all single cell was counted, and we divided the total number of single cell by the total number of spheres to obtain the relative volume of single sphere.

### Flow cytometry

On the sixth day of incubation, sphere cells are centrifuged at 800 rpm for 5 min, and the supernatant is discarded. The pelleted cells were digested with 0.25% trypsin EDTA (Life Technologies) and centrifuged again at 800 rpm for 5 min. Next, the supernatant was discarded and the cells were resuspended in 100 μL of PBS. Following this, 5 μL of the human anti-CD133-PE (1:50 dilution, Miltenyi Biotec) was added in the dark and the solution was incubated on ice for 30 min in the dark. Next, 500 μL of PBS was added to resuspend the cells, followed by centrifugation (800 rpm for 5 min), and the supernatant was discarded. Then, 200 μL of PBS was added to resuspend the cells, and expression of the cell surface marker CD133 was detected using flow cytometry (PAS, PARTEC, Germany).

### qRT-PCR

Total RNA was extracted using OMEGA (#R6934B). Equal amounts of RNA were reverse-transcribed to cDNA using the Revert Aid First Strand cDNA Synthesis Kit (Thermo Fisher Scientific, #K1622, Waltham, MA, USA). qRT-PCR analysis was performed using quantitative Ultra SYBR Mixture (Low ROX) (CWBIO, #CW2601), according to the manufacturer’s instructions. We used the RNA from SYT-SSX1 synovial sarcoma Paraffin tissues as a positive control. The data were interpreted using the 2^-△△CT^ method (Livak and Schmittgen), and gene expression levels were normalized against β-actin levels. The primer sequences were designed by Sangon Biotech, and were as follows: SYT 5′-CCAGCAGAGGCCTTATGGATA-3′ and SSX1 5′-GTGCAGTTGTTTCCCATCG-3′; β-actin forward (F): 5′-GAGCGGGAAATCGTCCGTGACATT-3′ and β-actin reverse (R): 5′-GATGGAGTTGAAGGTAGTTTCGTG-3′. The other primer sequences used for qPCR were as follows: SOX2 (F: 5′-CATCACCCACAGCAAATGACA-3′ and R: 5′-GCTCCTACCGTACCACTAGAACTT-3′); Nanog (F: 5′-CTAAGAGGTGGCAGAAAAACA-3′ and R: 5′-CTGGTGGTAGGAAGAGTAAAGG-3′); OCT4 (F: 5′-GCAGCGACTATGCACAACGA-3′ and R: 5′-CCAGAGTGGTGACGGAGACA-3′); LIF (F: 5′-CTGTTGGTTCTGCACTGGAA-3′ and R: 5′-CCCCTGGGCTGTGTAATAGA-3′); LIFR (F: 5′-AGCCTCAAGCAAAACCAGAA-3′ and R: 5′-TTGGCCTGAGGTCTGTAACC-3′); Ki-67 (F: 5′-CAGTACTCGGAATGCAGCAA-3′ and R: 5′-CAGTCTTCAGGGGCTCTGTC-3′); and β-actin (F: 5′-AATGTCGCGGAGGACTTTGAT-3′ and R: 5′-AGGATGGCAAGGGACTTCCTG-3′). qRT-PCR was performed using a 7500 Fast Real-Time PCR system.

### Cell treatment methods

To induce EMT, cells were seeded into 6-well plates and grown to 70–80% confluency in complete growth medium. Recombinant human TGF-β1 (R&D Systems, MN, USA) was reconstituted in 4 mM HCl containing 0.1% bovine serum albumin. Cells were then incubated in serum-free medium supplemented with TGF-β1 at concentrations of 0, 1, 5, and 10 ng/mL at 37 °C in a normal humidified atmosphere. Cells were harvested 36 h after treatment. CCK-8 assays were used to evaluate cell proliferation. All experiments were performed in triplicate and performed three times.

To inhibit EMT, SB431542 (Selleck Chemicals, Boston, USA), a TGF-β1 inhibitor, was reconstituted to a 10 mM stock solution in DMSO. Cells were incubated in serum-containing medium supplemented with 0, 1, 5, or 10 μM SB431542 at 37 °C in a normal humidified atmosphere. Cells were harvested 24 h after treatment. All experiments were performed in triplicate and repeated three times.

### Cell proliferation assay

We measured and evaluated the cell proliferation at different time points using the CCK-8 kit (Dojindo, Beijing, China), following the manufacturer’s protocol. Specifically, the cells of each of the above experimental groups were seeded in a 96-well plate at 6000 cells per well. After continuous incubation of cells in a standard environment, the optical density at 450 nm was measured at 0, 24, and 48 h using spectrophotometry (Bio-Rad, USA), followed by measurement of cell proliferation rate. The assays were conducted in triplicate and repeated at least three times.

### Statistical analysis

SPSS Statistics, Version 22.0 software and GraphPad Prism 8.0 were used to analyze the experimental data. The experimental data are presented as the means ± SD, and a *t*-test was used for statistical analysis. *P* < 0.05 was considered statistically significant.

## Results

### SYT-SSX1 promotes migration and invasion in SW982 cells

**Fig. 1:**
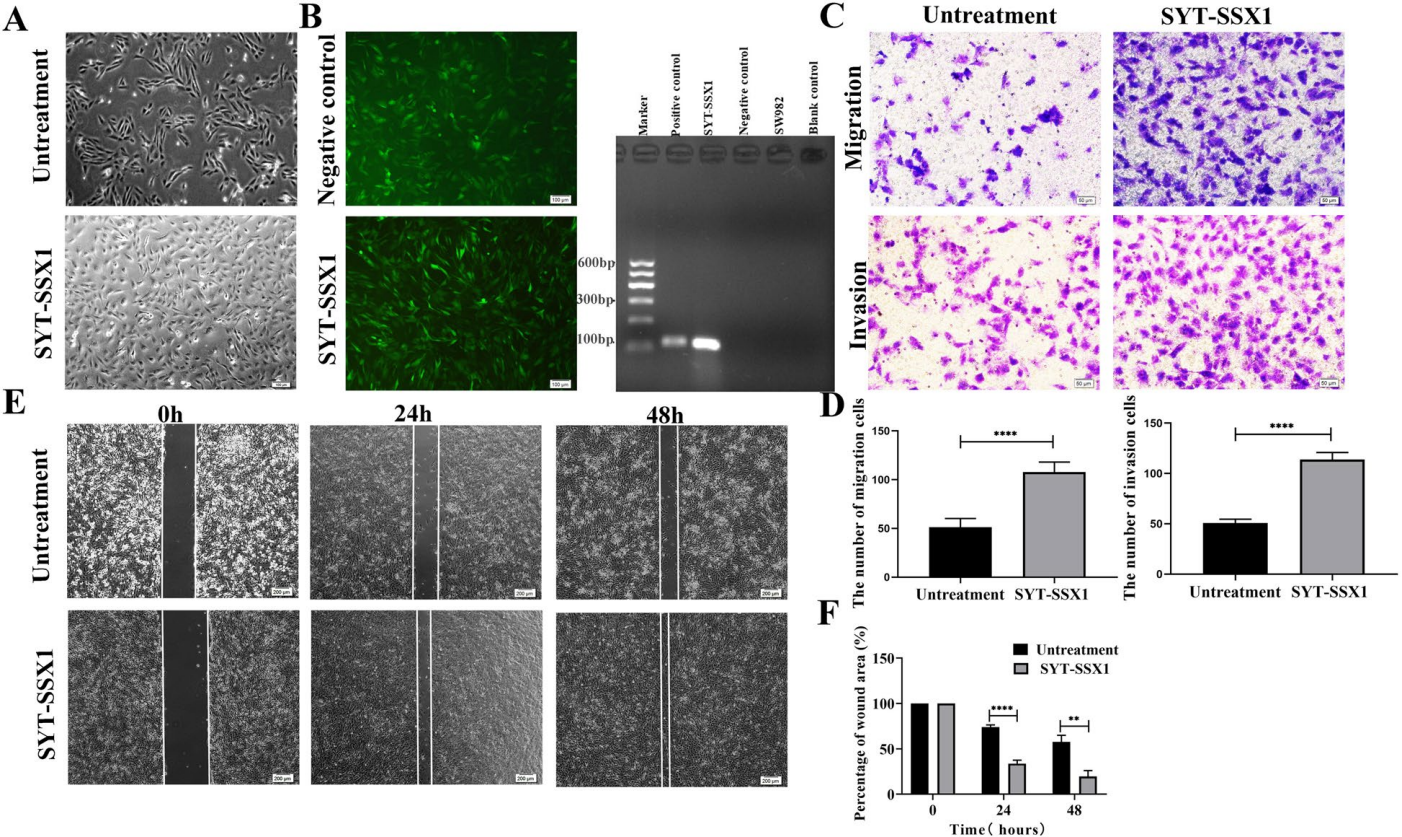
SYT-SSX1 induces cell invasion, and migration in SW982. **A** Morphological observation of cells transfected with SYT-SSX1 fusion gene for 48 h and untreated SW982 cells. **B** SW982 cells were transfected with GFP-tagged SYT-SSX1 lentivirus and negative control lentivirus to observe the fluorescent expression (Magnification: 100X). The *SYT-SSX1* fusion gene was detected using qPCR and the resulting PCR product was 118 bp. **C**-**D** Detection of the invasion and migration ability of SYT-SSX1 cells by transwell experiment. (E-F) Detection of the migration ability of SYT-SSX1 cells via a wound healing experiment. Images of the closing wound at 0, 24 h, and 48 h following the scratch (Magnification: 40X). Quantification of the wound closure area. Error bars represent the means ± SD.*P < 0.05, **P < 0.01, ***P < 0.001

We constructed the *SYT-SSX1* fusion gene in SW982 cells using lentivirus transduction. After 72 h of transduction, cells carrying the target gene were screened with 0.5 μg/mL puromycin to select for SYT-SSX1-overexpressing cell lines (named SYT-SSX1). After 48 h of transduction, the cells were observed to be fusiform or polygonal, with a large nucleus and clear cytoplasm, and appeared to be rapidly proliferating in comparison to the parental cells (Fig. 1A). SW982 cells overexpressing GFP-tagged SYT-SSX1 or GFP alone (as a negative control) were observed and assessed based on fluorescence intensity. Images were taken of fluorescent and non-fluorescent cells in the same field of view, and 5 fields were randomly selected; subsequently, the proportion of fluorescent cells was counted, and the average value was taken to obtain a transduction efficiency of 93.86% (0.9386 ± 0.02). After the successful screening of GFP-positive clones, RNA of SYT-SSX1 high-expressing cells, negative control lentiviral cells, and untreated SW982 cells was extracted, and the expression of *SYT-SSX1* fusion gene was detected via One Step RT-PCR. Our results showed that the *SYT-SSX1* fusion gene was present in the GFP-tagged SYT-SSX1 cells; moreover, the *SYT-SSX1* fusion gene was not detected in either in the negative control lentiviral cells or the untreated cells (SW982 cells). A single amplified band was located at 118 bp (Fig. 1B).

To compare the migratory and invasive abilities of the SYT-SSX1 cells and their parental SW982 cells, we performed transwell migration and invasion assays (Fig. 1C-D) as well as a wound healing assay (Fig. 1E-F). Notably, SYT-SSX1 cells had significantly higher rates of migration and invasion compared with the parent SW982 cells (*P* < 0.0001). The results from the wound healing assay also suggested that the migration ability of the SYT-SSX1 cells were significantly enhanced compared to the parent SW982 cells at 24 h and 48 h (*P* < 0.0001 and *P* = 0.002, respectively). These data suggest that the SYT-SSX1 cells are more malignant than parent SW982 cells.

### SYT-SSX1 enhances spheroid formation and maintains the stemness characterization

**Fig. 2:**
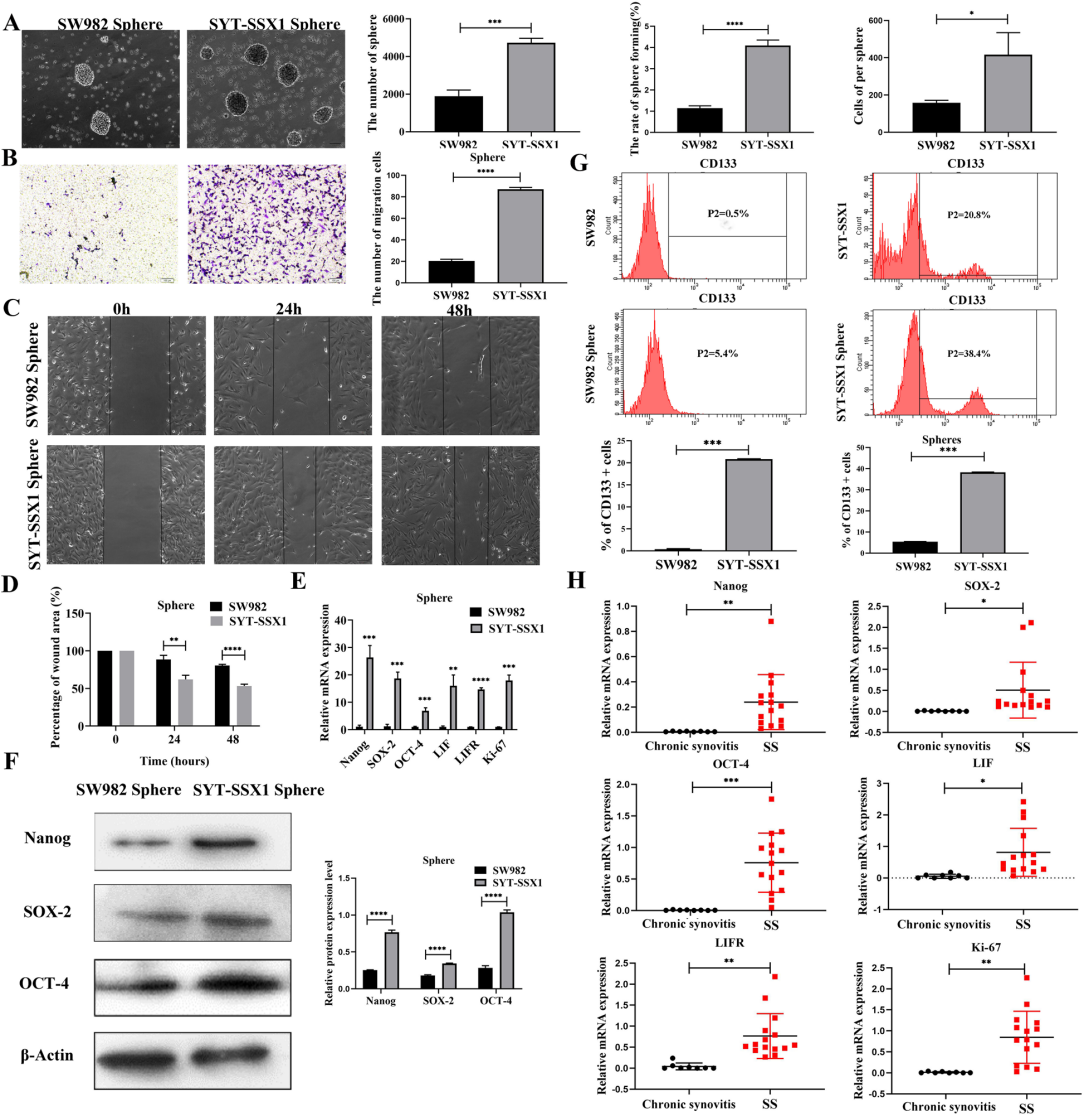
SYT-SSX1 enhances stem-like cell properties. **A** The number of cells, volume, and sphere-forming rates were calculated for SYT-SSX1 expressing cells and parental SW982 control cells. **B** Transwell assay showed that the migration ability of SYT-SSX1 sphere cells was stronger than that of SW982. **C**-**D** Enhanced migration ability of SYT-SSX1 sphere cells. Results from microscopy indicate the observations in the scratch experiment at 0, 24, and 48 h (Magnification: 100X). The scratch area was quantified. **E**-**F** qPCR and western blotting showed that cancer stem cell (CSC) markers (SOX-2, Nanog, and OCT-4), self-renewal factors (LIF and LIFR), and proliferation index (Ki-67) were highly expressed in SYT-SSX1 sphere cells compared with that of SW982. **G** Flow cytometry showed SYT-SSX1^sp^ cells highly expressed the CSC marker CD133; the protein expression level was significantly higher than SW982 sphere cells. **H** Fifteen cases of synovial sarcoma tissue and 8 cases of next to the tumor (control group) were examined using qRT-PCR to detect the expression of CSC markers, self-renewal factors, and proliferation index. All experiments were repeated at least three times. Error bars represent the means ± SD. β-actin served as an internal control. *P < 0.05, **P < 0.01, ***P < 0.001

CSC can enhance the aggressiveness and promote the metastasis of cancer cells. We generated cell spheres to produce a higher percentage of CSC-like populations. SW982 and SYT-SSX1 cells were cultured in a serum-free suspension culture for 72 h. Following 72 h, SYT-SSX1 cells showed many spheroid formations; the single spheroid cell was closely arranged had suspended growth. The spheroids were spherical or elliptical, whereas SW982 cells exhibited only partial sphere formation. Moreover, the number of SYT-SSX1 cells within their spheres was significantly higher (2.5-fold) than that of SW982 cells (*P* < 0.001). A single spheroid from the SYT-SSX1 (SYT-SSX1^sp^) line was larger than that of the SW982 cell line; the volume of single spheroid formed from SYT-SSX1 cells was significantly larger (2.1-fold) than that of SW982 single sphere (*P* < 0.05). The sphere forming rate of the SYT-SSX1 cells was significantly increased by 3.6-fold compared to those from the SW982 cells (*P* < 0.0001) (Fig. 2A).

After culturing the suspension sphere cells for 72 h in serum-free medium, the migration ability of the sphere cells was assessed using the transwell migration assay. We found that the number of SYT-SSX1 sphere cells (SYT-SSX1^sp^) that migrated was significantly higher than that of the SW982 sphere cells (SW982^sp^) (*P* < 0.0001; Fig. 2B). Next, we monitored how well SYT-SSX1^sp^ and parental SW982^sp^ cells migrated following a scratch after 24 and 48 h and measured percent wound closure. Our results suggested that the SYT-SSX1^sp^ cells were significantly faster than that of the parent SW982^sp^ cells at 24 h and 48 h (*P* = 0.005 and *P* < 0.0001, respectively). These data suggest that SYT-SSX1^sp^ cells are more migratory than parent SW982^sp^ cells (Fig. 2C-D).

The expression of the CSC markers (Nanog, SOX-2, and OCT-4), self-renewal factors (LIF and LIFR), and proliferation index (Ki-67) of the SYT-SSX1 sphere cells and the SW982^sp^ cells were detected via RT-PCR and WB analyses after 72 h of sphere culturing. The results showed that in SYT-SSX1^sp^ cells, the mRNA and protein expression levels of CSC markers (Nanog, SOX-2, and OCT-4), and the mRNA levels of the self-renewal factors (LIF and LIFR) and proliferation index (Ki-67) were significantly higher than those in the SW982^sp^ cells (*P* < 0.001; Fig. 2E-F). These data suggest that SYT-SSX1 promotes the expression of CSC markers at the mRNA and protein level in addition to promoting self-renewal and proliferation in SS sphere cells.

Next, we used flow cytometry to detect the expression of CSC markers in spheroid cells. For cells in the SW982^sp^ negative control group, the expression level of the surface marker CD133 was 5.4%. Contrastingly, compared with the SYT-SSX1 type high expression sphere negative control group, cells in the SYT-SSX1^sp^ group contained a markedly higher proportion of CD133 expression (38.4%) in comparison to the SW982^sp^ negative control group (Fig. 2G). These findings suggest that SYT-SSX1 can promote the expression of tumor stem cell protein markers to a certain extent.

To further examine the role of SYT-SSX1, we collected tissues from 15 patients with synovial sarcoma and 8 tissues from chronic synovitis or next to the tumor, and then detected mRNA levels of the CSC markers (Nanog, SOX-2, and OCT-4), self-renewal factors (LIF and LIFR) and proliferation index (Ki-67) using RT-PCR. Compared with the control tissues (chronic synovitis), the mRNA expression of SOX-2, Nanog, OCT-4, LIF, LIFR and Ki-67 was significantly higher in the synovial sarcoma tissue samples (*P* = 0.0070, *P* = 0.0487, *P* = 0.0002, *P* = 0.0110, *P* < 0.0001, and *P* = 0.0011, respectively) (Fig. 2H). Combined, our data indicate that SYT-SSX1 promotes the formation of sphere cells and maintains the stemness of SS cells while also enhancing the ability of SS sphere cells to self-renew, proliferate, and migrate.

### Influence of the TGF-β1/Smad pathway on the EMT phenomenon in SYT-SSX1 cells

**Fig. 3:**
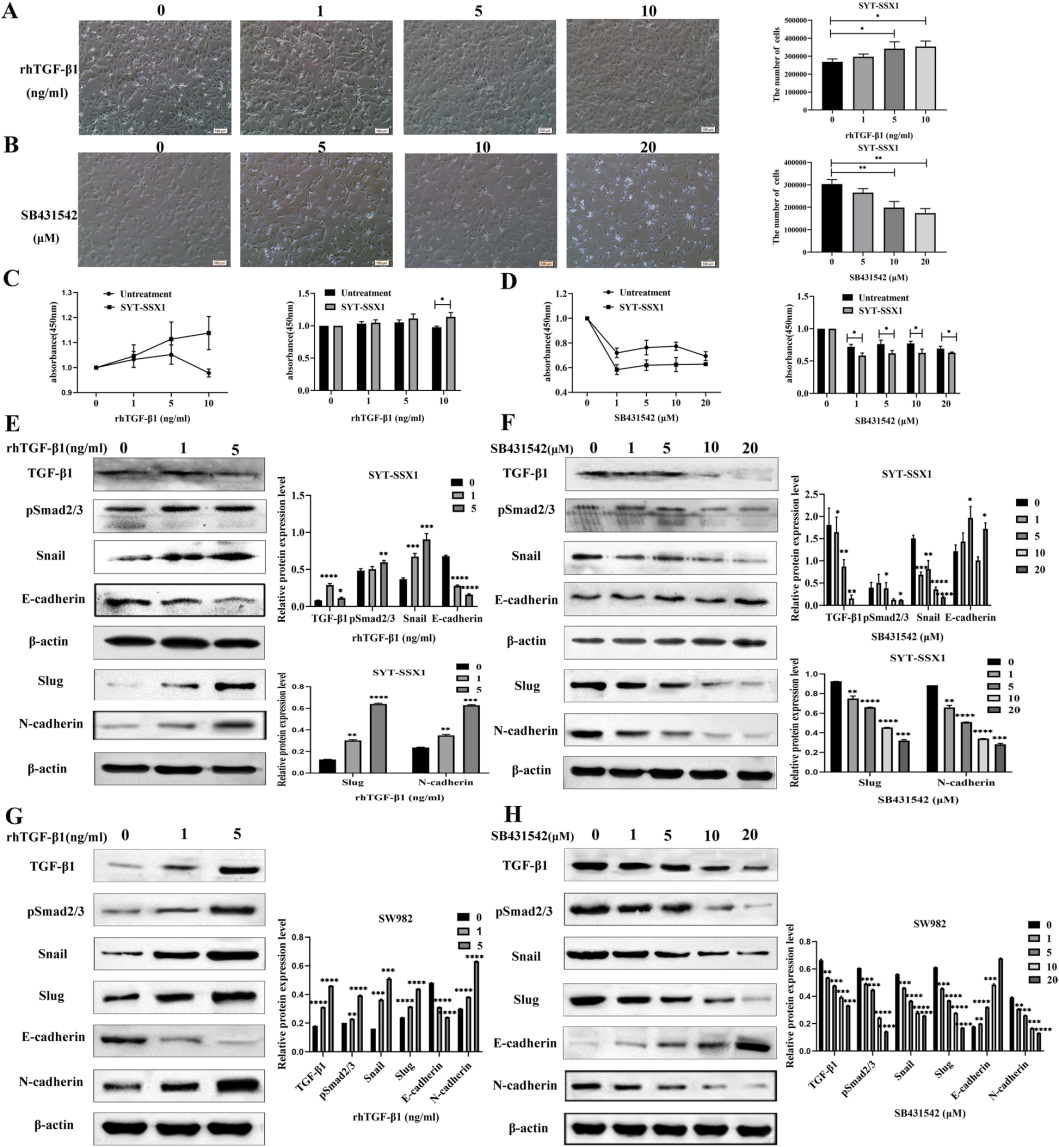
TGF-β1 enhances the proliferation and induces EMT in SYT-SSX1 cells. **A** Morphological observations of SYT-SSX1 cells following treatment with 0, 1, 5, or 10 ng/mL rhTGF-β1 for 36 h. **B** Morphological observations of SYT-SSX1 cells following treatment with 0, 5, 10, or 20 μM SB431542 for 36 h. **C** CCK-8 assay on SYT-SSX1 or untreated cells following rhTGF-β1 treatment at different concentrations for 36 h. **D** CCK-8 assay on SYT-SSX1 or untreated cells following SB431542 treatment at different concentrations for 36 h. **E** Following treatment with 0, 1, or 5 ng/mL rhTGF-β1 on SYT-SSX1 cells, western blotting was used to probe for TGF-β1, pSmad2/3, Snail, E-cadherin, Slug, and N-cadherin. **F** After treating with SB431542 (0, 1, 5, 10, and 20 μM) on SYT-SSX1 cells, western blotting was used to probe for TGF-β1, pSmad2/3, Snail, E-cadherin, Slug, and N-cadherin. **G** Following treatment with 0, 1, or 5 ng/mL rhTGF-β1 on SW982 cells, western blotting was used to probe for TGF-β1, pSmad2/3, Snail, and E-cadherin. **H** After treating with SB431542 (0, 1, 5, 10, and 20 μM) on SW982 cells, western blotting was used to probe for TGF-β1, pSmad2/3, Snail, and E-cadherin. All experiments were repeated at least three times. Error bars represent the means ± SD. β-actin served as an internal control. *P < 0.05, **P < 0.01, ***P < 0.001

To examine the effects of EMT on the activity of SYT-SSX1 cells via the TGF-β1/Smad pathway, we treated cells with 0, 1, 5, or 10 ng/mL rhTGF-β1 (a recombinant agent of the TGF-β1 signaling pathway). Following treatment with rhTGF- β1, we found that cell morphology changed from elongated to spindled and the number of cells gradually increased in a dose-dependent manner. Compared to the untreated condition, SYT-SSX1 cells treated with 5 ng/mL and 10 ng/mL of rhTGF-β1 had significantly higher cell counts (*P* = 0.038 and *P* = 0.013, respectively) (Fig. 3A). Further, we compared the proliferative ability of SYT-SSX1 and SW982 cells using the CCK8 assay. After 36 h of treatment with 10 ng/mL rhTGF-β1, proliferation was promoted in the SYT-SSX1 cells and was stronger than that of the untreated control (SW982 cells) group (*P* = 0.016) (Fig. 3C). Interestingly, we detected the expression of components from the TGF-β1 pathway (TGF-β1, pSmad2/3) and EMT-related proteins (Snail, Slug, E-cadherin, N-cadherin) in SYT-SSX1 cells and SW982 cells treated with different concentrations of rhTGF-β1 (0, 1, or 5 ng/mL) (Fig. 3E, Fig. 3G). The protein expression of TGF-β1, pSmad2/3, Snail, Slug and N-cadherin increased with 1 ng/mL and 5 ng/mL of rhTGF-β1, whereas the expression of E-cadherin decreased; specifically, the most prominent effect was observed in the 5 ng/mL rhTGF-β1-treated group in SYT-SSX1 cells.

Conversely, we treated the SYT-SSX1 cells with a TGF-β1 inhibitor, SB431542, at concentrations of 1, 5, 10, and 20 μM. Changes in cell morphology were observed every 12 h, and the total number of cells was counted after 36 h. When treated with SB431542, SYT-SSX1 cells showed a gradual elongation of the cell morphology and cell death became more pronounced with increasing concentrations (Fig. 3B). Compared to the untreated condition, SYT-SSX1 cells treated with 10 μM and 20 μM SB431542 had significantly lower cell counts (*P* = 0.006, *P* = 0.002, respectively). To examine the effect of inhibiting TGF- β1 on proliferation, SYT-SSX1 cells were treated with SB431542 (0, 1, 5, 10, and 20 μM) and monitored using the CCK8 assay. After 36 h of TGF- β1 inhibition, we found that SB431542 significantly inhibited the proliferation of SYT-SSX1 cells compared to the untreated group in a dose-dependent manner (*P* = 0.014, *P* = 0.028, *P* = 0.018, and *P* = 0.039; Fig. 3D). Compared with the control group, the expression of pSmad2/3, Snail, Slug and N-cadherin decreased, whereas the expression of E-cadherin increased under SB431542 treatment (1, 5, 10, 20 μM); notably, significant changes in expression levels were observed when cells were treated with 10 μM and 20 μM SB431542 (Fig. 3F, Fig. 3H). These results indicate that inhibition of the TGF-β1/Smad signaling pathway could inhibit cell growth and the EMT transformation of SYT-SSX1 cells.

### The TGF-β1/Smad signaling pathway enhanced the cancer cell stemness in SS cells

**Fig. 4:**
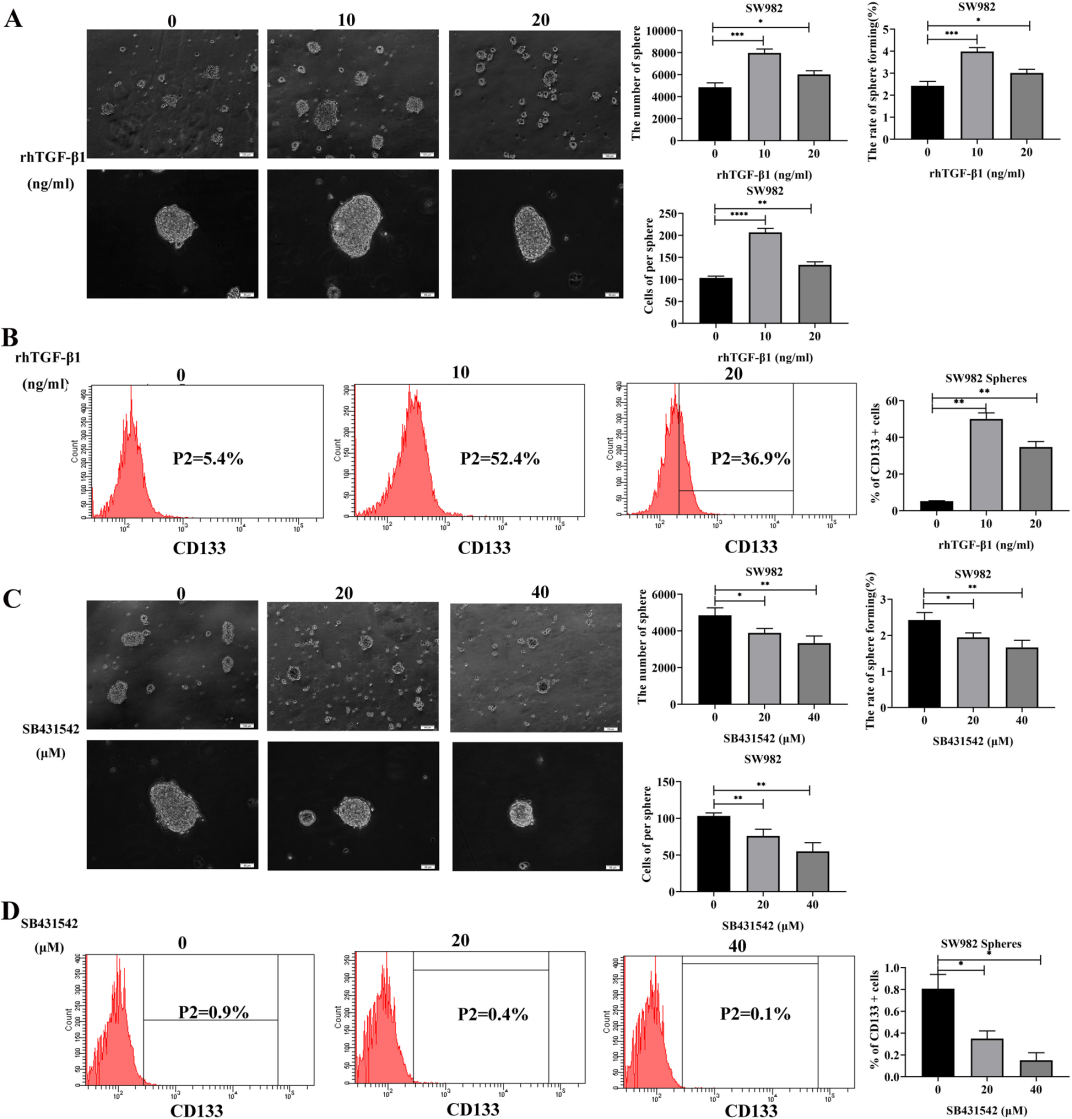
TGF-β1 affects cancer cell stemness in SW982 cells. **A** Morphological observations of sphere formation in SW982 cells following treatment with 0, 10, or 20 ng/mL rhTGF-β1 after 36 h. The number of cells, volume, and sphere-forming rates were calculated. **B** Flow cytometry was used to detect the expression of the CSC surface marker, CD133, on sphere cells of SW982 following rhTGF-β1 (0, 10 or 20 ng/mL treatment for 36 h). **C** Morphological observation of sphere formation in SW982 cells following treatment with 0, 20, or 40 μM SB431542 after 36 h. The number of cells, volume, and sphere-forming rates were calculated. **D** Flow cytometry was used to detect the expression of the CSC surface marker, CD133, on spheres of SW982 cells following SB431542 (0, 20 or 40 μM) treatment for 36 h. All experiments were repeated at least three times. Error bars represent the means ± SD. *P < 0.05, **P < 0.01, ***P < 0.001, ****P < 0.0001

To check whether the TGF-β1/Smad pathway affects SS tumor stem cell-like cells, SW982 cells were treated with either 0, 10, or 20 ng/mL rhTGF-β1 and changes in the morphology of the spheroids formed were monitored. Compared with the control group, the number (*P* = 0.0006, *P* = 0.0184), volume (*P* < 0.0001, *P* = 0.0033), and sphere-forming rate (*P* = 0.0006, *P* = 0.0184) of SW982^sp^ increased when treated with rhTGF-β1 at 10 and 20 ng/mL concentrations, respectively. The 10 ng/mL treatment group showed the most pronounced effects (Fig. 4A). These results indicate that rhTGF-β1, can enhance the sphere-forming ability of SW982 cells.

Next, we used flow cytometry to evaluate the expression levels of the CSC surface marker CD133. We determined that the percent expression of CD133 in the treatment groups (10 and 20 ng/mL rhTGF-β1) were 52.4 and 36.9%, respectively, whereas the percentage in the control group was 5.4% (Fig. 4B). The results indicate that rhTGF-β1can enhance the stemness of SW982^sp^ cells. Conversely, we treated SW982 cells to observe morphological changes of sphere formed under different concentrations of SB431542 (0, 20, and 40 μM). Compared with the control group, the number (*P* = 0.0253, *P* = 0.0099), volume (*P* = 0.0095, *P* = 0.0026), and sphere-forming rate (*P* = 0.0253, *P* = 0.0099) of SW982^sp^ treated with SB431542 at 20 μM and 40 μM, respectively, evidently decreased. Notably, the results observed for the 40 μM treatment group were the most drastic (Fig. 4C). These results show that use of the TGF-β1 signaling pathway inhibitor, SB431542, can inhibit the sphere-forming ability of SW982 cells. Furthermore, results from flow cytometry showed that the expression of CD133 in the treatment groups (20 and 40 μM SB431542) was 0.4 and 0.1%, respectively, whereas the percentage of CD133 in the control group was 0.9% (Fig. 4D). These results show that inhibiting the TGF-β1 signaling pathway with SB431542 can also inhibit the stemness of SW982^sp^ cells.

**Fig. 5:**
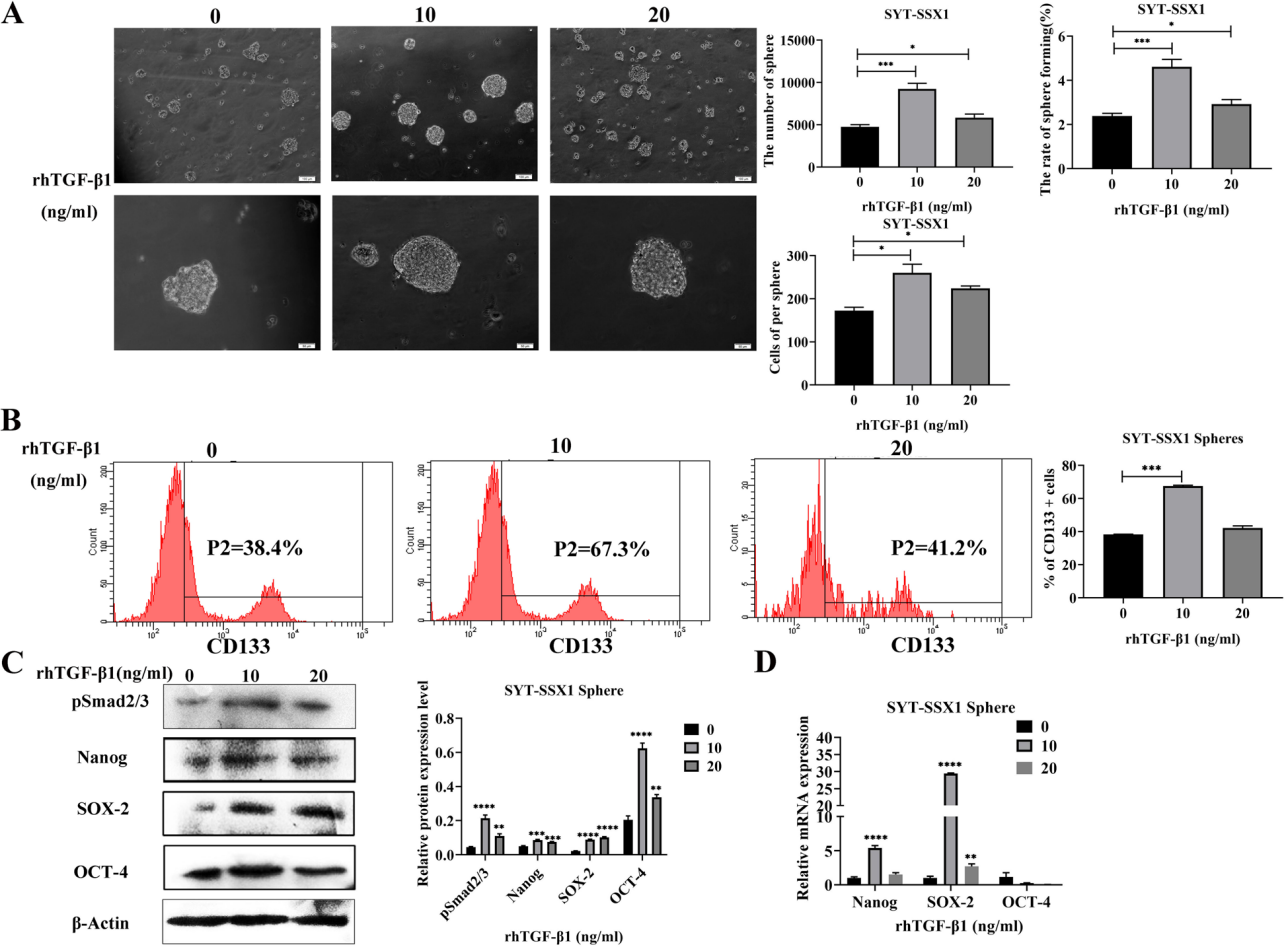
TGF-β1 enhances cancer cell stemness in SYT-SSX1 cells. **A** Morphological observations of sphere formation in SYT-SSX1 cells following treatment with 0, 10, or 20 ng/mL rhTGF-β1 for 36 h. The number and volume of spheres, and sphere-forming rate were calculated. **B** Flow cytometry was used to detect the expression of the CSC surface marker, CD133, on sphere cells of SYT-SSX1 following rhTGF-β1 (0, 10 or 20 ng/mL) treatment for 36 h. **C**-**D** Western blotting and qRT-PCR were used to detect the expression of the CSC surface markers Nanog, SOX-2, and OCT-4 on sphere cells of SYT-SSX1 following rhTGF-β1 (0, 10 or 20 ng/mL) for 36 h. All experiments were repeated at least three times. Error bars represent the means ± SD. β-actin served as an internal control. *P < 0.05, **P < 0.01, ***P < 0.001, ****P < 0.0001

To further assess the effects of TGF-β1 signaling on SYT-SSX1 generated spheres, we observed the morphological changes of sphere formation from SYT-SSX1 cells following treatment for 36 h with 0, 10, or 20 ng/mL rhTGF-β1. Compared with the control group, the number (*P* = 0.0004, *P* = 0.0193), volume (*P* = 0.0109, *P* = 0.0175), and sphere-forming rate (*P* = 0.0004, *P* = 0.0193) were significantly increased in SYT-SSX1^sp^ cells treated with 10 and 20 ng/mL rhTGF-β1, respectively; notably, the effects were the most significant when treated with 10 ng/mL rhTGF-β1 (Fig. 5A). These results show that promoting TGF-β1 signaling through rhTGF-β1 enhances the sphere-forming ability of SYT-SSX1^sp^ cells. Using flow cytometry, the expression rate of CD133 was 67.3 and 41.2% in the 10 and 20 ng/mL rhTGF-β1-treated SYT-SSX1^sp^ groups, respectively, whereas the rate in the control group (0 ng/mL rhTGF-β1) was 38.4% (Fig. 5B). Interestingly, the protein expression of the CSC markers (SOX-2, Nanog, and OCT-4) and pSmad2/3 of SYT-SSX1^sp^ was significantly increased following rhTGF-β1 treatment (10 and 20 ng/mL) for 36 h as determined using WB; notably, the change in the 10 ng/mL treatment group was the most significant (*P* < 0.001; Fig. 5C). When the SYT-SSX1^sp^ cells were treated with 10 ng/mL of rhTGF-β1, the mRNA expression of SOX-2 (*P* < 0.01) and Nanog (*P* < 0.05) significantly increased, whereas OCT-4 levels were decreased (Fig. 5D). These results suggest that rhTGF-β1 promotes the expression of the CSC markers at both the mRNA and protein level in SYT-SSX1^sp^ cells.

**Fig. 6:**
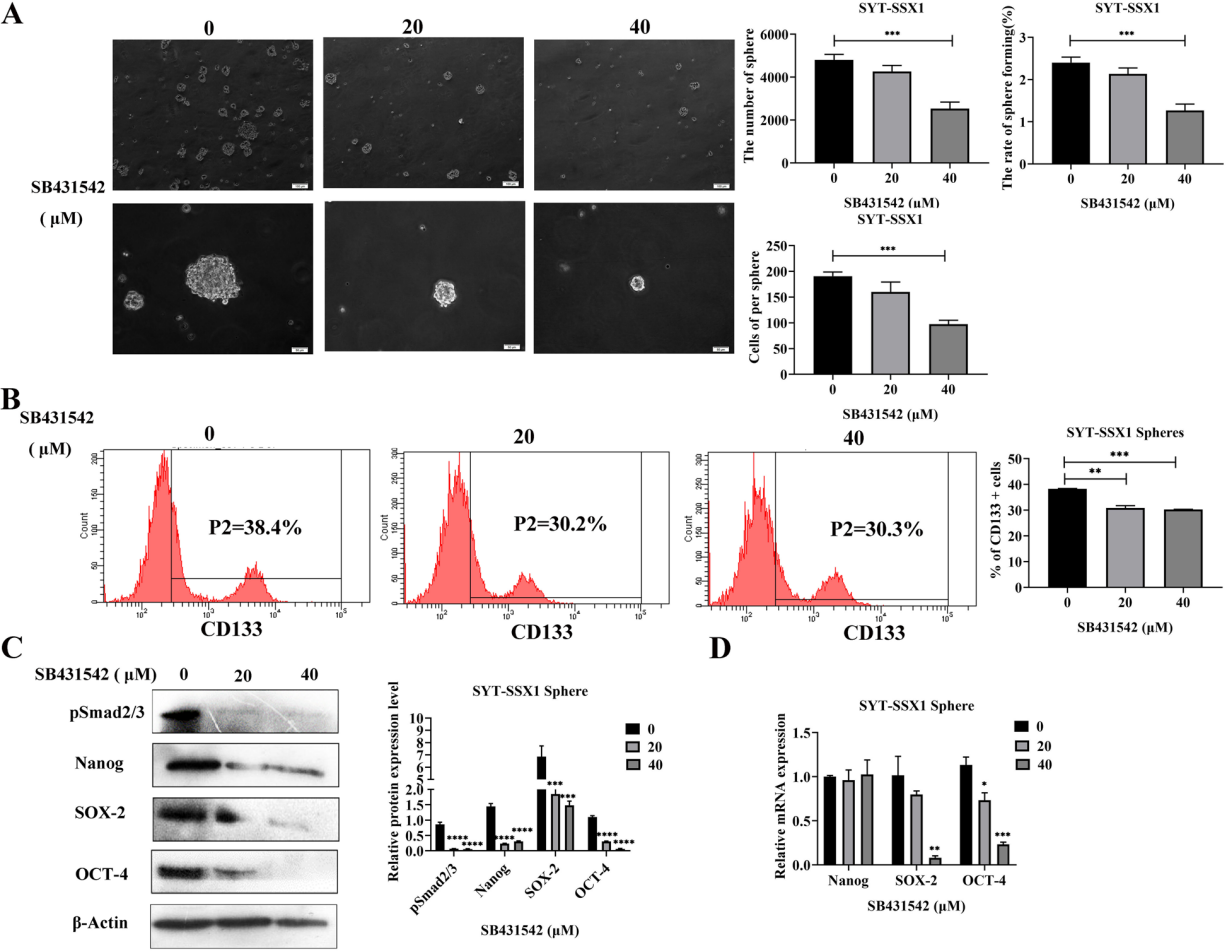
SB431542 suppresses cancer cell stemness in SYT-SSX1 cells. **A** Morphological observation of sphere formation in SYT-SSX1 cells following treatment with 0, 20, or 40 μM SB431542 after 36 h. The number and volume of spheres, and sphere-forming rate were calculated. **B** Flow cytometry was used to detect the expression of the CSC surface marker, CD133, on sphere cells of SYT-SSX1 following SB431542 (0, 20 or 40 μM) treatment for 36 h. **C**-**D** Western blotting and qRT-PCR were used to detect the expression of CSC surface markers on sphere cells of SYT-SSX1 following SB431542 (0, 20 or 40 μM) treatment for 36 h. All experiments were repeated at least three times. Error bars represent the means ± SD. β-actin served as an internal control. *P < 0.05, **P < 0.01, ***P < 0.001, ****P < 0.0001

Conversely, we treated SYT-SSX1 cells with increasing concentrations of SB431542 (0, 20, and 40 μM) for 36 h and observed the changes in spherical cell morphology. Compared with the control group, the cell number, volume, and sphere-forming rate were significantly reduced in SYT-SSX1^sp^ cells following treatment with 40 μM SB431542 (*P* < 0.001; Fig. 6A). These results show that the TGF-β1 signaling pathway inhibitor SB431542 can also block sphere formation in SYT-SSX1^sp^ cells. Next, using flow cytometry, we determined that the expression rate of CD133 in SYT-SSX1^sp^ cells was 30.2 and 30.3% following treatment with 20 and 40 μM SB431542, respectively, whereas the percent CD133 present was 38.4% in the untreated group (Fig. 6B). Following SB431542 treatment with 20 or 40 μM concentrations in SYT-SSX1^sp^ cells, the protein levels of the CSC markers (SOX-2, Nanog, and OCT-4) and pSmad2/3 were significantly decreased; interestingly, treatment with 40 μM S431542 showed the most significant difference (*P* < 0.0001; Fig. 6C). Moreover, the mRNA levels of SOX-2 and OCT-4 (*P* < 0.001) were significantly lower; however, the mRNA expression of Nanog did not change (Fig. 6D). Collectively, these results show that SB431542 can inhibit the expression of tumor stem cell-related proteins and mRNA transcripts in SYT-SSX1^sp^ cells to a certain extent. As a result, the TGF-β1/Smad signaling pathway enhances CSC in SYT-SSX1^sp^ cells.

## Discussion

The relationship between CSCs and the *SYT-SSX* fusion gene is a question of urgent interest in SS. Recent reports have indicated that the *SYT-SSX* fusion gene is an important predictor of the clinical prognosis of patients with SS. The 5-year metastasis-free survival rate of SYT-SSX1 patients was 42%, whereas that of SYT-SSX2 patients was 89% [23]. The literature shows that there is an association between the SYT-SSX fusion type and histological subtype. All cases of BSS have SYT-SSX1 fusion, while MFSS has *SYT-SSX1* and *SYT-SSX2* fusion genes [24]. These results suggest that the different fusion genes and the morphology of SS may contribute to EMT through various molecular genetic changes. The overexpression of fusion genes may be associated with stem cell identity, which raises the possibility that these stemness-related genes induce CSC-like tumor phenotypes and aggressive tumor behavior [2]. In this study, we demonstrated that SYT-SSX1-positive cells overexpressed OCT-4, Nanog, SOX2, LIF, LIFR, and Ki-67, which are thought to form the interrelated autoregulatory circle and maintain the transcriptional activity required for pluripotent stemness. OCT-4, Nanog, and SOX-2 are known to be expressed only in specific human cancer types [25, 26]. Our previous study showed a similar expression profile for CSC markers (CD133, CD29, CD44, Nestin, and ALDH1) in all 20 clinical SS samples, suggesting that SS may be one of the tumors inherently possessing stem cell-like traits [27].

In SS, the expression of stem cell transcription factor SOX2 and its histone H3K27me3 triggered by mutations in the SS18-SSX driver caused tumor development [28]. When the *SYT-SSX* fusion gene in SS was silenced, researchers found that SS cells differentiated into pluripotent mesenchymal stem cells, indicating the *SYT-SSX* fusion gene as the basis for pluripotent stem cells to differentiate into tumors [3]. Luisa et al. found that the *SYT-SSX1* fusion gene induces and maintains the stemness of mesenchymal stem cells by activating polycomb group proteins [29]. Therefore, in this study, we successfully cultured SYT-SSX1 and SW982 spheres with a serum-free suspension culture method. Our results showed that compared with SW982 cells, the sphere formation abilities were significantly stronger in SYT-SSX1-positive cells, including the size, number, and rate of sphere formation. Further, the migration ability of SYT-SSX1^sp^ cells was also stronger than that of SW982^sp^ cells, indicating that the *SYT-SSX1* fusion gene can promote the proliferation, self-renewal, and migration of SS stem cell-like cells.

Previously, we showed that the TGF-β1 signaling pathway promotes SS cell EMT-like phenomenon, cell movement, and cell invasion [30]. In this study, we found that the TGF-β1 signaling pathway can regulate the *SYT-SSX1* fusion gene and promote the proliferation of SS cells. The TGF-β1 signaling cascade also regulates the *SYT-SSX1* fusion gene promotion of EMT transformation, suggesting that SYT-SSX1 confers SS with tumor stem cell-like characteristics via TGF-β1 signaling. Mani et al. found that EMT can cause breast epithelial cells to have tumor stem cell-like characteristics [31]. At the same time, other studies have shown that metastatic cancer cells may exhibit CSC phenotypes after undergoing EMT. For example, after breast cancer metastasis, in sporadic pleural effusion, breast cancer cells are rich in CSC-like cell populations with high CD44 expression and low CD24 expression [32].

TGF-β belongs to the cytokine superfamily. The overexpression of TGF-β in various types of human cancers is associated with tumor progression, metastasis, angiogenesis, and poor prognosis. TGF-β is an effective driver of cancer progression by inducing EMT, which can guide cancer cells to dedifferentiate and acquire characteristics similar to CSCs [33]. The TGF-β signaling pathway plays an important role in regulating cell growth, differentiation, and migration, thereby participating in tumor progression [34]. The combination of TGF-β and the TGF-βII receptors (TGFβ-RII) initiates TGF-β signaling, which phosphorylates TGFβ-RI that in turn phosphorylates Smad2/3 to form Smad2/3/4 complexes, thereby activating downstream signals [35]. Bhola et al. found that chemotherapy-induced TGF-β caused tumor recurrence through IL-8 in CSCs, as TGF-β enhanced the stemness of the breast cancer cells; moreover, TGF-β type I receptor kinase inhibitors prevented the development of drug-resistant CSCs [36]. Zhang et al. found that inhibiting the TGF-β1 pathway not only reduced the expression of HIF-1a, but also inhibited the dedifferentiation of osteosarcoma cells induced by hypoxia and reduced the self-renewal ability of tumor stem cells [37]. Therefore, the TGF-β1 signaling pathway may promote SS stem cell characteristics through the *SYT-SSX1* fusion gene, providing new ideas for targeted gene therapy of SS.

After treating SYT-SSX1^sp^ cells with increasing concentrations of rhTGF-β1, the number of sphere cells and volume reached its maximum, indicating that rhTGF-β1 promotes the self-renewal ability of SYT-SSX1 type SS stem cell-like cells. This finding is consistent with the research results from Wang et al. They found that TGF-β increases the number of spheroids of breast cancer cells, which is a characteristic endowed by stem cells [38]. This study found that when rhTGF-β1 was at an appropriate concentration (i.e., 10 ng/mL), the expression levels of the CD133, SOX-2, Nanog, and OCT-4 proteins increased. However, when the concentration of rhTGF-β1 (20 ng/mL) continued to increase, the protein expression levels of CD133, SOX-2, Nanog, and OCT-4 decreased. Regarding this phenomenon, Park suggested that low concentrations of TGF-β and the binding protein MUSASHI-2 work together to promote the proliferation of hematopoietic stem cells [39]. When rhTGF-β1 was at an appropriate concentration (10 ng/mL), the mRNA expression levels of SOX-2 and Nanog increased, but the expression levels of OCT-4 mRNA decreased, indicating that SOX-2 and Nanog are relatively sensitive to rhTGF-β1 treatment in SS. You et al. treated Huh-7 cells with 10 ng/mL of rhTGF-β1 [40]. After 48 h, FACS analysis revealed that the number of cells expressing the surface marker CD133 increased. Additionally, it was verified at the protein level that TGF-β1 could promote the expression of the CD133 protein. Studies have shown that LSK cells exhibit a biphasic response to TGF-β2. When the concentration of TGF-β2 was high, the growth of LSK cells was inhibited, but when the concentration of TGF-β2 was low, it promoted the proliferation of LSK cells [41]. This observation is consistent with our results, indicating that a certain concentration of rhTGF-β1 can enhance the stemness of SYT-SSX1 type SS stem cell-like cells.

After treating SYT-SSX1 sphere cells with increasing concentrations of SB431542, we observed that the number and volume of the sphere cells gradually decreased. Other researchers, including Xie, have added SB431542 to CD34 + CD31+ progenitor cells and found that the number of red blood cells was significantly reduced in the early stage of red blood cell differentiation [42]. This finding is consistent with our results, indicating that SB431542 can inhibit the self-renewal ability of SYT-SSX1 type SS stem cell-like cells. Our experiments using flow cytometry and WB showed that as the concentration of SB431542 increased, the protein levels of CD133, SOX2, Nanog, and OCT-4 decreased. Furthermore, as shown using qRT-PCR, as the concentration of SB431542 increased, SOX-2 and OCT-4 mRNA levels decreased, but Nanog mRNA levels did not change significantly. Chen et al. found that, compared with undifferentiated iPSCs/ESC cells, the expression of SOX-2 and OCT-4 in cells treated with SB431542 decreased, but the expression of Nanog in iPSCs cells did not change, which is consistent with our results [43]. Altogether, these findings indicate that a certain concentration of SB431542 inhibited the stemness of SYT-SSX1 type SS stem cell-like cells.

Results from this study also indicate that the different CSC markers vary among the different tissues, and that the sensitivity to various drug treatments are also different, all of which is affected by many factors. In this study, we preliminarily explored the role of the *SYT-SSX1* fusion gene in SS stem cell-like cells and its correlation with the TGF-β1/Smad signaling pathway. Further in vivo experiments are needed to study the mechanism of action between them, as CSCs are also affected by PI3K/mTOR, Notch, and Wnt/β-catenin signaling, as well as other signaling pathways, EMT, the microenvironment, microRNA, and various other factors. Future studies should focus on whether the effect of these factors on the stemness of SS stem cell-like cells is related to the *SYT-SSX* fusion gene, or whether it has a synergistic effect with the TGF-β1/Smad signaling pathway to jointly regulate the stemness of SS stem cell-like cells. Exploring these possible relationships will provide new strategies for the treatment of CSC.

## Conclusions

To summarize, the *SYT-SSX1* fusion gene promotes invasion, migration, and enhances stem-cell-like characteristics in SS cells by inducing TGF-β1/Smad signaling. These findings reveal an effective way to potentially improve the prognosis of patients with SS by eliminating the characteristics of cancer stem cells during treatment. However, the effects on tumor growth when blocking the TGF-β1/Smad pathway in vivo were not validated. The relationship among EMT, CSC-related markers, SYT-SSX1 expression, and prognosis in clinical pathological specimens were also not explored, and still requires further investigation. Addressing such limitations could serve as a basis for future studies.

## Supplementary Information

**Additional file 1.**

## Data Availability

The datasets used and/or analyzed during the current study are available from the corresponding author on reasonable request. All data generated or analyzed during this study are included in this published article [and its supplementary information files].
