# Supplementary material for: SYT-SSX1 enhances the invasiveness and maintains stem-like cell properties in synovial sarcoma via induction of TGF-β1/Smad signaling
Source: BMC Cancer. 2022 Feb 12;22:166. doi: 10.1186/s12885-022-09229-5 (PMC8841078; doi:10.1186/s12885-022-09229-5)
Supplement: Supplementary file 1 — Additional file 1. [file 12885_2022_9229_MOESM1_ESM.zip › Supplementary materials of the amended manuscript BMC cancer.pdf]

SYT-SSX1 enhances the invasiveness and maintains stem-like cell properties in synovial sarcoma via induction of TGF-β1/Smad signaling

Shuang-Shuang Dong[1*], Yong-Lai He[3]*, Zi-Han Liu[5], Ya-Lan Huang[6], Ning Wang[1], Zhen Zhang[1], Zhong Li[1], Mei Er •Tu He Ta Mi Shi[1], Xiao Feng[1], Qing Yao[1], Hong Zou[1], Jian-Ming Hu[1], Li-Juan Pang[1], Feng Li[1,4#] and Yan Qi[1,2#]

1 Department of Pathology, Shihezi University School of Medicine & the First Affiliated Hospital to Shihezi University School of Medicine, Shihezi 832002, Xinjiang, China.

2 Department of Pathology, Central People's Hospital of Zhanjiang & Zhanjiang Central Hospital, Guangdong Medical University.

3 Department of ICU, Central People's Hospital of Zhanjiang & Zhanjiang Central Hospital, Guangdong Medical University.

4 Department of Pathology, Beijing Chaoyang Hospital, Capital Medical University, Beijing, China.

5 Department of Pathology, The Affiliated Hospital of Qingdao university, Qingdao, China.

6 Department of Pathology, Suining Central Hospital, Suining, Sichuan, China.

*Contributed equally

#Correspondence to: Dr Yan Qi Department of Pathology, Shihezi University School of Medicine & the First Affiliated Hospital to Shihezi University School of Medicine, Shihezi 832002, Xinjiang, China, North 2 road, Shihezi, Xinjiang 832002, P.R. China; Department of Pathology, Certral People's Hospital of Zhanjiang & Zhanjiang Central Hospital, Guangdong Medical University. Email: qiyanyan-1998@163.com

Dr Feng Li Department of Pathology, Beijing Chaoyang Hospital, Capital Medical University, Beijing, China. Email: lifeng7855@126.com

Supplementary materials:

The original bands related to the WB of Figure 2F (Nanog, OCT-4, SOX-2 and β-Actin), Figure 3E (TGF-β1, pSmad2/3, Snail, E-cadherin and β-Actin), Figure 3F (TGF-β1, pSmad2/3, Snail, E-cadherin and β-Actin), Figure 5C (pSmad2/3, Nanog, OCT-4, SOX-2 and β-Actin), Figure 6C (pSmad2/3, Nanog, OCT-4, SOX-2, β-Actin) are included in the supplementary material.
